# Cardiomyopathy With Preexcitation and Conduction Abnormalities in a Child

**DOI:** 10.1002/joa3.70191

**Published:** 2025-09-12

**Authors:** Gorav Sharma, Ansh Goswami, Siddharthan Deepti, Neeraj Parakh, Sudheer Kumar Arava

**Affiliations:** ^1^ Department of Cardiology All India Institute of Medical Sciences New Delhi India; ^2^ Department of Pathology All India Institute of Medical Sciences New Delhi India

**Keywords:** cardiomyopathy, case report, conduction abnormalities, fasciculoventricular pathway, LDB3 mutation

## Abstract

An 11‐year‐old boy presented with dilated cardiomyopathy in association with a fasciculoventricular pathway and sinus node and atrioventricular conduction abnormalities. Whole exome sequencing revealed a novel variant of uncertain significance in LIM domain‐binding protein 3 (LDB3) which has not been heretofore described. This variant was predicted to be deleterious by the computational prediction tools: Polyphen2 and SIFT. The mutated allele was heterozygous in the patient. Segregation analysis revealed that his father carried the same variant in heterozygous form. The case is reported for its rarity.

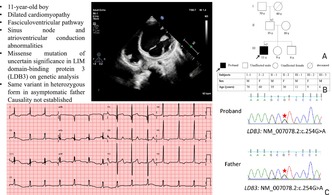

An 11‐year‐old boy presented with decompensated heart failure. Transthoracic echocardiogram showed severe biventricular dysfunction with an ejection fraction of 25% and left ventricular end‐systolic and end‐diastolic dimensions of 48 mm and 56 mm. No significant left ventricular hypertrophy was present.

The 12‐lead electrocardiogram (ECG) was notable for the presence of ectopic atrial rhythm with negative *p*‐waves in leads II, III, and aVF (Figure 1). The PR interval was 80 milliseconds. Ventricular preexcitation was noted, with slight slurring of the QRS upstroke in several leads. Though preexcitation was not maximal, the pattern suggested the presence of a right superoparaseptal atrioventricular bypass tract with isoelectric delta wave in lead V1, positive delta wave in leads I, II, aVL, and aVF, and precordial QRS transition in lead V3. A 24‐h ambulatory ECG recording, however, showed the same degree of subtle preexcitation during sinus rhythm, with no change with varying heart rates (Figures S1 and S2).

**FIGURE 1:**
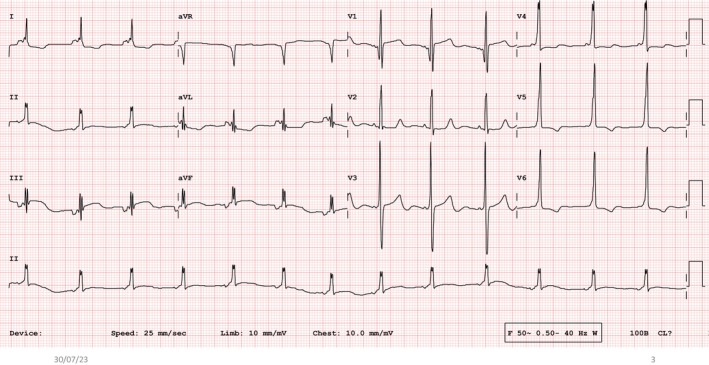
Twelve‐lead electrocardiogram showing ectopic atrial rhythm with negative *p*‐waves in leads II, III, and aVF. The PR interval is 80 milliseconds. Mild ventricular preexcitation is noted with isoelectric delta wave in lead V1, precordial RS transitional zone in lead V3 and positive delta wave in leads I, II and aVL.

An electrophysiologic study was performed after stabilization. Quadripolar catheters were positioned in the high right atrium, the His bundle (HB) region, and the right ventricular apex, and a deflectable decapolar catheter into the coronary sinus. The earliest ventricular activation was noticed at surface QRS and the HB region. Two sharp deflections (H and H′) were observed in the His bundle channel between atrial (A) and ventricular (V) electrograms (Figure 2A). The AH, HH′, and HV intervals were measured to be 40 ms, 30 ms, and 24 ms, respectively. With incremental atrial pacing and atrial extrastimulation from the high right atrium, there was separation of A and H, proving that H was not a part of the fragmented atrial electrogram (Figure 2B). Atrial pacing led to gradual prolongation of the AH interval, but the HH′ and HV intervals remained constant. At an atrial pacing cycle length of 350 ms, there was loss of preexcitation with the appearance of typical complete right bundle branch block (RBBB) (Figure 2C). It was noted that the H′ potential was no longer visible, and the HV interval was prolonged to 76 ms, with corresponding prolongation of the PR interval to 140 ms. Atrial extrastimulus testing showed atrioventricular block with HV block at 350 ms, suggestive of infrahisian pathology (Figure 2B). A 12‐mg bolus of intravenous adenosine produced atrioventricular block, with the junctional escape beat having the same degree of preexcitation, with no change in the HV interval (Figure 2D). Ventricular pacing and ventricular extrastimulation showed decremental ventriculoatrial conduction with concentric atrial activation. Tachycardia was not inducible with any of the pacing maneuvers performed.

**FIGURE 2:**
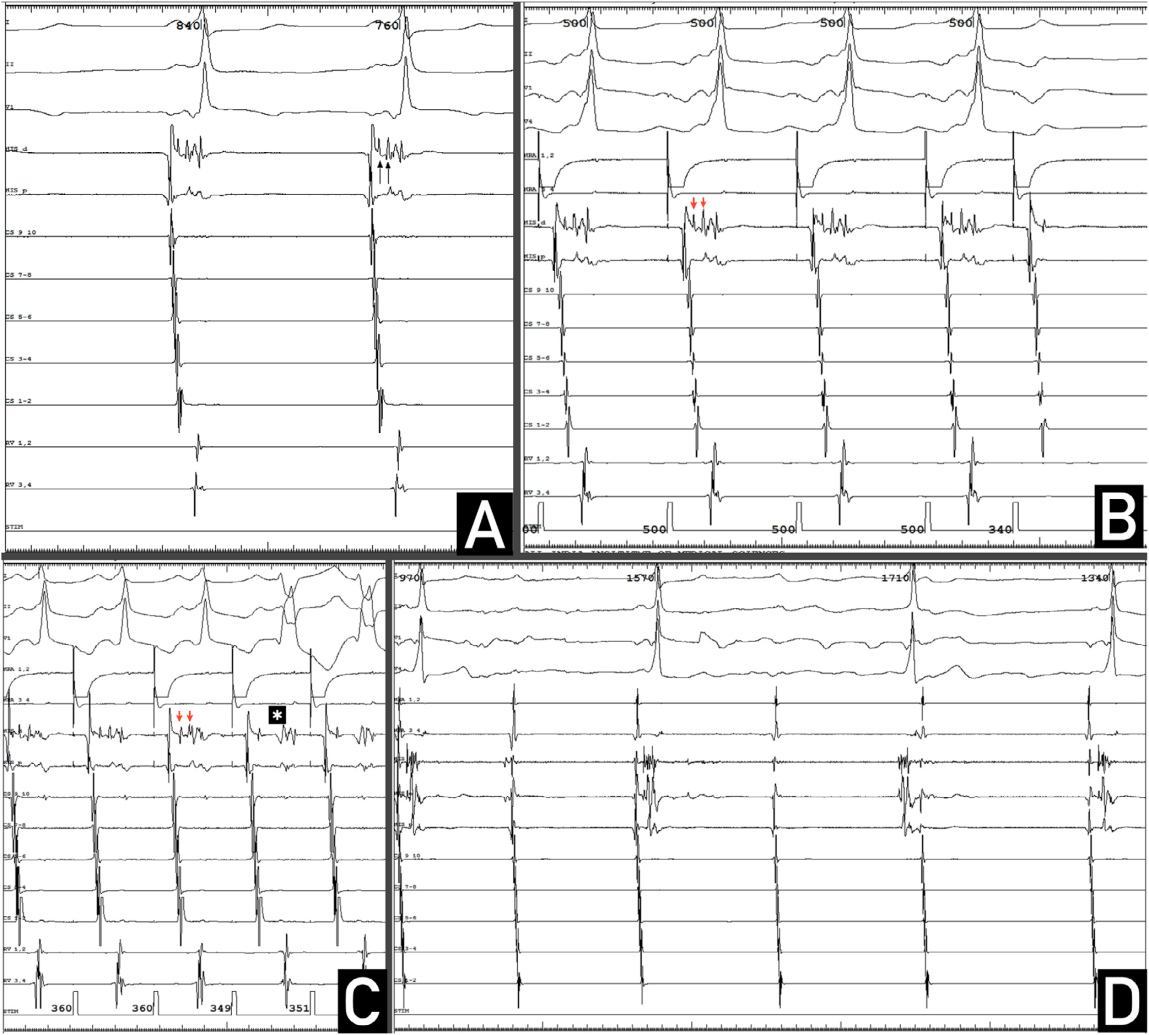
(A) Baseline intracardiac electrograms showing two sharp deflections (H and H′) indicated with black arrows in the His bundle channel between atrial (A) and ventricular (V) electrograms. (B) Atrial extrastimulation after a train from high right atrium leads to gradual prolongation of AH interval, but the HH′ and HV intervals remain constant. Atrioventricular block with HV block happens with atrial extrastimulus at 350 ms, suggestive of infrahisian pathology. (C) Loss of preexcitation with the appearance of typical complete right bundle branch block at an atrial pacing cycle length of 350 ms. There is loss of H′ indicated by asterisk with loss of preexcitation and the appearance of RBBB thereby suggesting that the H′ might represent the fasciculoventricular pathway potential. (D) Atrioventricular block with the junctional escape beat having the same degree of preexcitation with no change in HV interval after a 12‐mg bolus of intravenous adenosine.

Fixed preexcitation at varying heart rates in sinus as well as ectopic atrial rhythm along with the earliest ventricular signal at the distal His bundle suggested the presence of a fasciculoventricular bypass tract. This was further supported by dependence on atrioventricular nodal conduction and the same degree of preexcitation with junctional ectopy [1].

The intracardiac electrograms were notable for the presence of a distinctive H′ potential which likely represented the fasciculoventricular pathway potential so that the H′V interval was very short at 24 ms and remained constant at all rates but disappeared with the loss of preexcitation when the effective refractory period of the pathway is reached at the pacing cycle length of 350 ms. It was notable because of the conduction delay from the His bundle (H) to the fasciculoventricular pathway. The infrahisian conduction abnormality was evident from HV block on atrial extrastimulation. Loss of preexcitation was associated with the appearance of complete RBBB. The pathway was, in fact, responsible for natural resynchronisation.

There also appeared to be underlying sinus node dysfunction with the presence of an ectopic low atrial rhythm at times. Thus, the diagnosis of fasciculoventricular accessory pathway (FVP) with sinoatrial and infrahisian conduction abnormalities was established on electrophysiologic study.

The simultaneous presentation with dilated cardiomyopathy, fasciculoventricular pathway, and conduction abnormalities engendered suspicion for inherited forms of preexcitation and cardiomyopathy. Whole exome sequencing was done on Illumina Novaseq 6000 platform, which led to the identification of a missense mutation NM_007078.2:c.254 G>A in the LDB3 gene in the proband encoding for LIM domain‐binding protein 3. This variant was predicted to be deleterious by the computational prediction tools: Polyphen2 and SIFT. The mutated allele was heterozygous in the patient. The variant's pathogenicity has not been confirmed by any functional study to date; hence, it was categorized as a variant of uncertain significance. Segregation analysis was performed using Sanger sequencing, which revealed that his father carried the same variant in heterozygous form (Figure 3).

**FIGURE 3:**
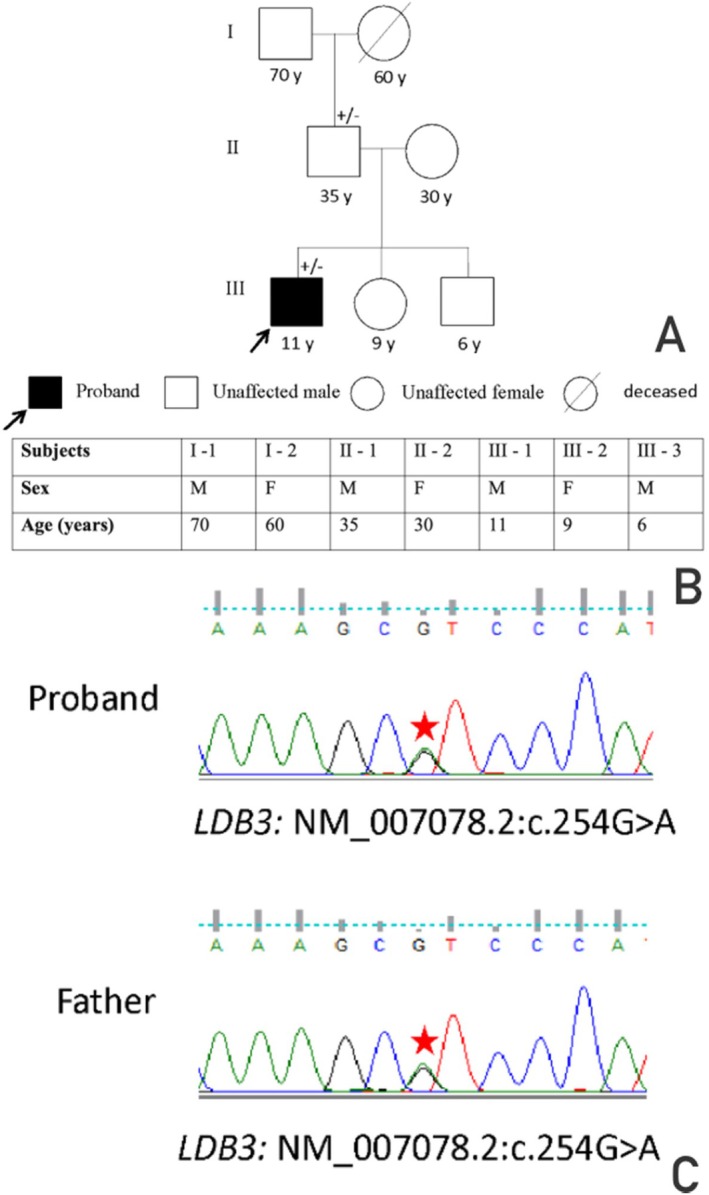
(A) The pedigree chart for the proband's family and the segregation of LDB3 mutations (+/indicates heterozygous, −/− indicates wild type); (B) Detailed characteristic features of patient III‐1 and his family members. (C) Chromatogram obtained with Sanger sequencing showing that the proband and his father carry the LDB3 gene variant (NM_007078.2:C.254 G>A) in heterozygous form.

Fasciculoventricular pathways have been described in association with inherited cardiomyopathies like PRKAG2 syndrome and Danon disease [2, 3]. Recognition of inherited preexcitation associated with cardiomyopathy is important for prognostication and avoidance of inappropriate therapies for the accessory pathway which never causes tachyarrhythmia. In fact, in the present case, the pathway was actually responsible for intrinsic resynchronization with underlying RBBB.

This report is the first description of a fasciculoventricular pathway with cardiomyopathy in the presence of a heretofore unreported missense mutation in the gene encoding for LIM domain‐binding protein 3 (LDB3). The causality cannot be confirmed because of the uncertain significance of the variant and its presence in an unaffected carrier. This protein is involved in maintaining the stability of the *Z*‐discs within the sarcomeres. Mutations in the LDB3 gene have been reported in cases of dilated and myofibrillar myopathies [4]. One study has reported testing of the LDB3 gene in the case of left ventricular noncompaction (LVNC) with Wolff‐Parkinson‐White syndrome [5]. There are no published reports on the role of LDB3 in conduction abnormalities.

To conclude, fasciculoventricular pathways rarely occur in inherited forms of cardiomyopathies and may be associated with sinus and atrioventricular nodal conduction disturbances in this setting. This report is the first description of a LDB3 variant in a patient with these electrophysiologic abnormalities.

## Conflicts of Interest

The authors declare no conflicts of interest.

## Supporting information


**Figure S1:** Ambulatory ECG recording showing the same degree of mild preexcitation during sinus rhythm at rates of 67 bpm (panel A) and 160 bpm (panel B).

**Figure S2:** A cartoon showing the relationship between the conduction system and the fasciculoventricular pathway in this case. The probable locations of the H and H′ signals are indicated. The right bundle branch block is represented by the curved black line.

## References

[1] E. B. Sternick , L. M. Gerken , M. O. Vrandecic , et al., “Fasciculoventricular Pathways: Clinical and Electrophysiologic Characteristics of a Variant of Preexcitation,” Journal of Cardiovascular Electrophysiology 14 (2004): 1057–1063. 10.1046/j.1540-8167.2003.03206.x 14521658

[2] E. B. Sternick , A. Oliva , L. M. Gerken , et al., “Clinical, Electrocardiographic, and Electrophysiologic Characteristics of Patients With a Fasciculoventricular Pathway: The Role of PRKAG2 Mutation,” Heart Rhythm 8, no. 1 (2011): 58–64. 20888928 10.1016/j.hrthm.2010.09.081

[3] S. Jhaveri , J. Herber , K. Zahka , G. J. Boyle , E. V. Saarel , and P. F. Aziz , “Arrhythmias and Fasciculoventricular Pathways in Patients With Danon Disease: A Single Center Experience,” Journal of Cardiovascular Electrophysiology 30, no. 10 (2019): 1932–1938. 31240821 10.1111/jce.14049

[4] M. Vatta , B. Mohapatra , S. Jimenez , et al., “Mutations in Cypher/ZASP in Patients With Dilated Cardiomyopathy and Left Ventricular Non‐Compaction,” Journal of the American College of Cardiology 42, no. 11 (2003): 2014–2027. 14662268 10.1016/j.jacc.2003.10.021

[5] N. K. Lakdawala and M. M. Givertz , “Dilated Cardiomyopathy With Conduction Disease and Arrhythmia,” Circulation 122, no. 5 (2010): 527–534. 20679582 10.1161/CIRCULATIONAHA.109.892240 PMC3027355

