# Supplementary figures and images for: Cardiomyopathy With Preexcitation and Conduction Abnormalities in a Child
Source: J Arrhythm. 2025 Sep 12;41(5):e70191. doi: 10.1002/joa3.70191 (PMC12432268; doi:10.1002/joa3.70191)

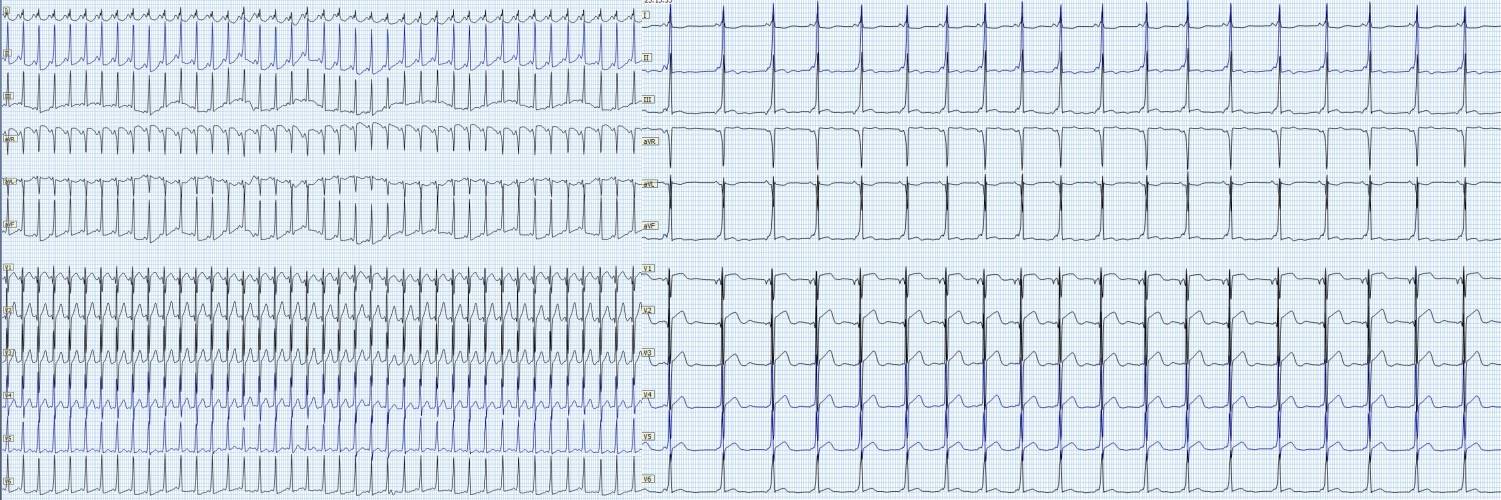

Supplement: Supplementary file 1 — Figure S1: Ambulatory ECG recording showing the same degree of mild preexcitation during sinus rhythm at rates of 67 bpm (panel A) and 160 bpm (panel B). [file JOA3-41-e70191-s001.jpg]

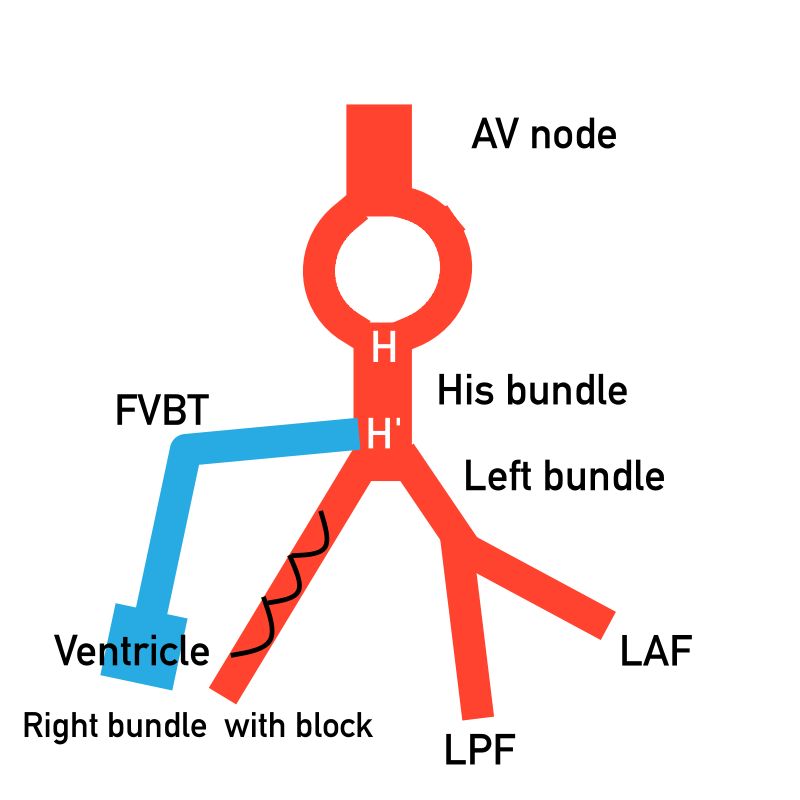

Supplement: Supplementary file 2 — Figure S2: A cartoon showing the relationship between the conduction system and the fasciculoventricular pathway in this case. The probable locations of the H and H′ signals are indicated. The right bundle branch block is represented by the curved black line. [file JOA3-41-e70191-s002.png]
